# Biomarker-based diagnosis of mild cognitive impairment due to Alzheimer’s disease: how and what to tell. A kickstart to an ethical discussion

**DOI:** 10.3389/fnagi.2014.00041

**Published:** 2014-03-19

**Authors:** Corinna Porteri, Giovanni B. Frisoni

**Affiliations:** ^1^Bioethics Unit, IRCCS San Giovanni di Dio FatebenefratelliBrescia, Italy; ^2^Laboratory of Epidemiology, Neuroimaging and Telemedicine, IRCCS San Giovanni di Dio FatebenefratelliBrescia, Italy; ^3^Geneva University Hospitals and University of GenevaGeneva, Switzerland

**Keywords:** prodromal Alzheimer’s disease, informed consent, diagnosis disclosure, translational research, bioethics guideline

## Abstract

New criteria for the diagnosis of Alzheimer’s disease (AD) based on biomarker results have recently been developed and are currently undergoing extensive validation. The next few years may represent a time window where the diagnostic validity of biomarkers will be studied in highly specialized research settings. Biomarkers results will be used to direct clinical diagnosis and, whenever appropriate, therapy and management. This piece aims to stimulate discussion by identifying the ethical challenges involved in the use of biomarkers to make a diagnosis of mild cognitive impairment due to AD and disclose it to patients. At the individual level, these challenges are related to (i) the ethical appropriateness of implementing an ecological diagnostic research protocol, (ii) the related informed consent process, and (iii) the diagnostic disclosure. We justify the ethical legitimacy of implementing a research diagnostic protocol by referring to the respect of patients’ subjectivity and autonomy, and we suggest guidelines for informed consent development and diagnostic disclosure. All of the above points are discussed in light of the unique features of AD, currently scanty treatment options, and knowledge and uncertainties regarding the diagnostic value of biomarkers.

## INTRODUCTION

The collection of evidence regarding the validity of biomarkers for disease detection is a dynamic process that will remain on-going until sufficient evidence has accumulated to satisfy the restrictive standards for widespread routine clinical use. New criteria for the diagnosis of Alzheimer’s disease (AD) based on biomarker data have been developed by an International Working Group in 2007 (Dubois et al., 2007,^2010^) and the National Institute on Aging and Alzheimer’s Association task force in 2011 (Albert et al., 2011). These criteria posit that AD can be diagnosed at the mild cognitive impairment (MCI) – or predementia/prodromal – stage through the assessment of so-called core biomarkers, including medial temporal atrophy on high-resolution magnetic resonance imaging (MRI), temporoparietal hypometabolism on fluorodeoxyglucose positron emission tomography (FDG-PET), Abeta42 and tau in the cerebrospinal fluid (CSF), and increased cortical uptake of amyloid ligands on PET. Preliminary but consistent evidence has been collected regarding the diagnostic validity of core AD biomarkers, i.e., their ability to detect subjects with memory impairment who have a high likelihood of exhibiting AD pathology and therefore progressing to dementia.

The authors of the new criteria and the scientific community at large advise that validation studies are needed for a more accurate estimate of the biometric features of biomarkers (sensitivity, specificity, positive predictive value, and negative predictive value). Moreover, the criteria have not been operationalised, i.e., it has not yet been established how biomarkers should be combined into a diagnostic algorithm, and measurement procedures, norms, and normality thresholds have not yet been standardized. To date, Amyvid^TM^ (Florbetapir F 18 injection) PET ligand has been approved by US and European agencies and Vizamyl^TM^ (Flutemetamol F18 injection) by US FDA for β-amyloid plaque density imaging in adult patients with cognitive impairment.

With this imaging protocols, a negative scan result greatly reduces the likelihood that a patient’s cognitive impairment is due to AD, while a positive scan enhances the likelihood of but does not establish a definitive diagnosis of AD.

The evidence required for confirmation of biomarker validity will be collected in highly specialized research settings through standardization of collection and measurement practices as well as collection of empirical data on the diagnostic accuracy of individual biomarkers and the incremental accuracy of combined biomarkers. In this phase, which might last a few years, ecological diagnostic research protocols will need to be developed and carried out that leverage on biomarker results. Thus, biomarkers will be collected, a judgment of normality/abnormality will be made based on pre-defined thresholds, the results will be compounded into a judgment of presence or absence of MCI due to AD, and the latter judgment will be delivered to the patient. The research aspect of the protocol will consist of controlling the procedures and outcomes of each of the above steps.

The related ethical issues deserve to be discussed. At the individual level, these issues relate to (i) whether implementing a diagnostic research protocol is ethically appropriate in the first place, and, if so, (ii) the development of a pertinent informed consent process and (iii) the appropriate diagnostic disclosure. The aim of this paper is to stimulate discussion in the community by indicating the ethical challenges involved in using biomarkers to make and disclose a diagnosis of MCI due to AD to patients in the clinical practice, and suggest possible operational guidelines.

## THE ETHICAL APPROPRIATENESS OF IMPLEMENTING A DIAGNOSTIC RESEARCH PROTOCOL

When facing the possibility of diagnostic use of biomarkers, the first ethical question relates to the appropriateness of implementing a diagnostic research protocol in which patients are offered access to a biomarker-based diagnosis. This is a major issue due to: the experimental nature of the procedure; the pre-disability stage of the disorder which implies the predictive nature of the diagnostic communication; the current lack of effective prevention and treatment options; the concern about “catastrophic reactions”; and the risk of stigmatization and misuse of diagnostic results by third parties (such as employers and insurance agencies; Gauthier et al., 2011; Karlawish, 2011; Prvulovic and Hampel, 2011). The answer to this fundamental question may be found by delving into patients’ requests. A typical case illustration might help to better clarify the point.

Patients with suspected MCI due to AD are generally (self-)referred to a medical service for memory problems, requesting specialist consultation. Clinical and cognitive assessments may reveal preservation of daily function associated with significant episodic memory impairment. Secondary causes of cognitive impairment (depression, iatrogenic effects, brain tumor, cerebrovascular disease, and others) are excluded. The patient is typically given a syndromic diagnosis of amnestic MCI (Petersen et al., 1999). Lacking access to biomarker assessment, the “diagnosis” of MCI is the only possible outcome of the consultation, and the patient is informed accordingly. However, it should be stressed that MCI is not a disease, in that it can be underlined by a number of diseases, including AD. It is well known that between one half and two thirds of MCI patients have AD, while the rest do not have progressive conditions and can even improve (Bennett et al., 2002). Thus, the communication of a “diagnosis” of MCI is uninformative to the patient, and it can even increase confusion about health status (Whitehouse et al., 2004).

Academic memory clinics aiming to validate diagnostic biomarkers have two options: (i) a purely scientific research protocol, in which biomarker data are collected for the purpose of biomarker standardization and validation, but patients are given no feedback on biomarker results; or (ii) an “ecological” diagnostic research protocol, in which biomarker data are collected for biomarker standardization and validation and patients are given feedback on biomarker results as well as an etiologic diagnosis. In the first case, data will be analyzed in an aggregate manner and no result will be studied with regard to the individual patient, while in the second case, data will also be analyzed on an individual basis to make a diagnosis. Importantly, case (i) excludes case (ii), but not *vice versa*.

At the present state of knowledge, both offering and not offering the opportunity to take part in a diagnostic research protocol has ethical implications, and the pros and cons need to be balanced (**Table 1**).

**Table 1:** Pros and cons of implementing a diagnostic research protocol based on Alzheimer’s biomarker assessment in MCI patients.

Cons	Pros
–Experimental nature of biomarker assessment	–Robust scientific evidence has demonstrated the diagnostic value of biomarkers
–Pre-disability stage of the disorder and predictive nature of the diagnosis	
–Lack of effective prevention and treatment options	–Respect for the patient’s wish to know what is going on in his/her brain
–Concern about “catastrophic reactions”	–Ability to plan for the future
–Risk of stigmatization and misuse of diagnostic results by third parties

The boundary between translational research and clinical practice can indeed be fuzzy, especially in the case of disorders with a pathogenesis that is still unclear and disorders for which effective treatments are still not available (Porteri et al., 2010). Thus, despite the fact that biomarkers are currently under study to ensure their validation and do not yet meet the strict requirements for use in routine practice, the careful use in a clinical context of research results that have already reached a sufficiently high degree of reliability can be in the patient’s interest more than choosing not to use them until full evidence is collected.

The strong ethical reason in favor of offering patients the opportunity to take part in a diagnostic research protocol is that patients refer to memory clinics with a medical problem asking for a diagnosis, and the use of biomarkers enhances physicians’ ability to make a more accurate diagnosis. The main ethical justifications for offering participation in a diagnostic research protocol include the respect for the patients regarded as autonomous subjects entitled to ask, know, and make decisions about their health and life; and the fact that biomarker assessment can provide incremental information. Currently, biomarker assessment can only be performed in a research context, and accepting the risks attached to a diagnosis that is made before the biomarker-based diagnosis is a routine procedure. Furthermore, a biomarker-based diagnosis can help patients plan for the future in terms of caregiving, health care choices, and financial and legal decisions, but does not provide immediate increased possibilities for treatment and prevention.

At this point, how to offer the subject the opportunity for a biomarker-based diagnosis is key. Respect for patient autonomy requires that the patient is given all information to consolidate his/her wish to know and is afforded the ability to change his/her mind and autonomously choose the best option.

## INFORMED CONSENT PROCESS FOR A BIOMARKER-BASED DIAGNOSIS

Given the above context, the informed consent process is a key element in biomarker assessment and deserves specific attention to ensure that the subject is fully able to understand the information regarding the diagnostic research protocol and to decide if he/she really wishes to undergo the evaluation and receive the results. Clarification of the issues at hand from the very beginning of the biomarker assessment process is mandatory to avoid damage and to maximize patient benefit. The experimental nature of the procedure makes the process of informed consent even more critical than it is in clinical routine practice, as the risks/benefits ratio is likely to be higher for procedures that have not been fully validated. Patients need to be aware of the experimental nature of the diagnostic examinations and of the value of the results that can be expected. In particular, the relatively uncertain nature of the diagnosis due to the need for further validation of the biomarkers should be explained in a realistic and understandable way to avoid confusion and misunderstandings.

We suggest that the information provided to patients should include the following essential elements (**Table 2**):

**Table 2:** Key elements of the informed consent process.

(a) The research nature of the diagnostic protocol (b) What the subject will be asked if he/she chooses to participate (c) What the individual subject can expect (d) The uncertainty of the diagnosis of MCI due to AD (e) The personal benefit of receiving the diagnostic results (f) The right to privacy (g) Non-participation or withdrawal from the research project (h) The option of sharing the result with the family (i) The option to not receive the diagnostic results

### (A) THE RESEARCH NATURE OF THE DIAGNOSTIC PROTOCOL

The subject needs to be aware of the research nature of the diagnostic protocol, i.e., the fact that a biomarker-based diagnosis is not yet part of clinical routine practice and biomarker validity is still under study. The physician will explain that the scientific community believes that biomarkers are potentially useful for making a more accurate and informed etiologic diagnosis than one of MCI. In particular, the scientific community anticipates that biomarker results can be used to discriminate between people who will and will not develop Alzheimer’s dementia. The physician will refer to previous studies showing that an individual biomarker abnormality has a high predictive value (Hansson et al., 2006; Mattsson et al., 2009) for the development of Alzheimer’s dementia within 5 years. The physician will explain that data are incomplete regarding the diagnostic accuracy of individual biomarkers and the incremental accuracy of combined biomarkers; however, it is quite likely that two or more abnormal markers will significantly enhance the predictive power (Frisoni et al., 2010; Prestia et al., 2013).

### (B) WHAT THE PATIENT WILL BE ASKED IF HE/SHE CHOOSES TO PARTICIPATE

The physician will explain what each biomarker examination entails, taking into account risks, burden, and discomfort associated with the procedures.

### (C) WHAT THE INDIVIDUAL SUBJECT CAN EXPECT

The physician will provide information on what can be expected from the study in terms of results that are relevant to the individual participant. Using a variable combination of biomarkers, a diagnosis of MCI due to AD could be made for the individual patient if the test results are positive. Alternatively, the diagnosis of AD could be excluded in those who test negative for the relevant biomarkers. The possibility that the results will not be informative also needs to be mentioned.

### (D) THE UNCERTAINTY OF THE DIAGNOSIS OF MCI DUE TO AD

The uncertainty of the diagnosis of MCI due to AD needs to be emphasized. On the one hand, the diagnostic validity of the biomarkers is highly probable, but it requires final demonstration in multiple large representative cohorts. This implies that the diagnostic use of biomarkers does not allow the formulation of a final diagnosis but rather a diagnosis with a high degree of confidence, particularly when the biomarkers are used together and the results are concordant. On the other hand, the diagnosis is expressed in terms of the probability rather than the certainty of developing Alzheimer’s dementia within five years from the first assessment.

### (E) THE PERSONAL BENEFIT OF RECEIVING THE DIAGNOSTIC RESULTS

The physician will explain to the subject the potential benefits arising from the disclosure of the diagnosis. These benefits include allowing those involved to make decisions on significant matters and to plan for the future. On the other hand, as far as a cure is concerned, the utility of knowing the diagnosis in advance is debatable at the present moment and is a matter of subjective judgment. No effective disease modifier is available for AD. Current drugs (i.e., cholinesterase inhibitors and memantine) can provide symptomatic relief in a number of patients but cannot delay disease progression. Unfortunately, these drugs, along with cognitive training and rehabilitation, for which there is still no indication of any significant benefit (Bahar-Fuchs et al., 2013), are the only real options today. It is reasonable to assume that starting treatments at a very early stage of the disease can result in better success both in terms of biological changes and clinical improvement (Gauthier and Leuzy, 2010; Prvulovic and Hampel, 2011); however, this hypothesis needs to be proven in clinical trials with new drugs.

### (F) THE RIGHT TO PRIVACY

The subject needs to be aware that every effort will be made to keep personal data confidential, according to the human being’s right to respect for private life and to local legislation. This will guarantee that third parties, such as employers or insurance agencies, will not be able to access subjects’ data and use these data against the subjects’ interests.

### (G) NON-PARTICIPATION OR WITHDRAWAL FROM THE RESEARCH PROJECT

The subject needs to be aware that he/she is not obliged to take part in the research diagnostic protocol and is free to withdraw from the protocol at any time, without having to provide a reason. The subject also needs to know that this decision will not in any way affect the relationship with the physician, although it will prevent the use of biomarkers to aid the diagnostic procedure.

### (H) THE OPTION OF SHARING THE RESULT WITH THE FAMILY

The subject will be offered the option of sharing**the information regarding the biomarker assessment as well as the disclosure of the diagnosis with a family member. The subject will also have the opportunity to delegate a family member to receive the examination results.

### (I) THE OPTION TO NOT RECEIVE THE DIAGNOSTIC RESULTS

The subject could be offered the opportunity to take part in the research protocol while deciding at the same time not to receive the experimental diagnostic results. In any case, he/she will retain the option of changing his/her mind throughout the biomarker assessment process and during medical follow-up.

Some of the above elements (a, c, d, e) are specific to the case under discussion, some are common to informed consent for research protocols in general (b, f, g), and others are common to diagnostic disclosure in general (h, i). All are essential elements that should be part of the information provided to the subject within a patient–physician relationship. The clinician should be able to adapt the information to the particular subject according to the subject’s specific circumstances, educational background, and ability to understand. The breadth of information that the physician will provide will need to be tailored to the individual patient’s ability to understand, ensuring that the subject will be offered the maximum level of information complexity he/she can retain. The verbal communication of information by the clinician is paramount and requires plenty of time to explain and answer questions and the ability to speak simply while not denying that there are unsolved issues together with the sensitivity to understand the subject’s expressed and unexpressed wishes.

The proposal relies on current knowledge and needs to be revised as new scientific evidence becomes available.

## DIAGNOSTIC DISCLOSURE OF MCI DUE TO AD

The disclosure of the diagnosis of Alzheimer’s dementia is difficult and complex even when the diagnosis is based on widely validated and internationally employed criteria. Guidelines for the disclosure of the diagnosis emphasize that telling the patient the truth in a sensitive way and avoiding unnecessary despair should be the usual practice to allow preservation of the patient’s quality of life and to play an active role in planning the future (Alzheimer Europe, 2009; Alzheimer’s Association, 2011).

Nevertheless, the guidelines are general, and there is wide variability in physicians’ attitudes and behaviors (Bamford et al., 2004; Werner et al., 2012); thus, practices based on local cultural values and preferences often prevail. In the case of the diagnosis of MCI due to AD based on the revised criteria, diagnostic disclosure is made even more difficult because the validity of these criteria is still under study (Porteri et al., 2010; Visser et al., 2012; Gauthier et al., 2013). Further elements of complexity for the physician relate to the generally greater awareness of patients with MCI compared to those with AD and the frequent lack of a family member with whom to share the burden of communication.

As a general rule, if a patient decides to undergo the diagnostic research protocol after all the information is provided, he/she is already aware of what can be expected as a diagnostic result at the end of the examinations. Nevertheless, additional issues need to be taken into consideration in the diagnostic disclosure of MCI due to AD (**Table 3**).

**Table 3:** Issues to be considered in the diagnostic disclosure of MCI due to AD.

– The therapeutic privilege in situations of clinical uncertainty – Cases that justify delayed diagnostic disclosure – The role of the subject’s family

### THE THERAPEUTIC PRIVILEGE IN SITUATIONS OF CLINICAL UNCERTAINTY

The element of “clinical uncertainty” is currently a peculiarity of the diagnosis of MCI due to AD. Apart from this element, with regard to the reasons for and against diagnosis communication, the disclosure of MCI due to AD does not differ from the disclosure of Alzheimer’s dementia in a stage when the patient has preserved reasoning and judgment. Indeed, Alzheimer’s dementia can be diagnosed in patients with global cognitive performance in the MCI range. The core of the physician’s disclosure is in fact the future development of dementia, i.e., disability, in a context of progressive cognitive and functional deterioration. Several reasons for and against the disclosure of an AD diagnosis are reported in the literature and experienced in clinical practice. The most important reasons for disclosing the diagnosis to patients involve the patient’s “right to know the truth” and the need to allow the patient to plan for the future. The principle of respect for autonomy plays a major role in decision-making with respect to disclosure. The most important reasons against disclosing the diagnosis of AD to patients are related to concerns about harming the patient and the intention to protect the patient from potentially adverse psychological reactions, including anxiety, depression, catastrophic thinking, suicidal ideation, and suicide. The principle of non-maleficence/beneficence plays a major role in deciding against full disclosure.

We suggest that the concept of therapeutic privilege be seriously taken into consideration in the disclosure of MCI due to AD. The concept applies to situations in which full preservation of both the principle of autonomy and the principle of beneficence seems to be impossible and the doctor, caught between the need to inform the patient and the wish to ensure the patient’s well-being by minimizing suffering, feels the obligation to renounce full disclosure to safeguard the patient’s well-being (Richard et al., 2010). Referring to the therapeutic privilege seems even more pertinent in biomedical research than in medical practice, given the different degree of uncertainty that further complicates the concept of the “right to know the truth.”

### CASES THAT JUSTIFY DELAYED DIAGNOSTIC DISCLOSURE

Patients who, even after adequate treatment, show symptoms of anxiety and depression and subjects without family support are subjects for whom the therapeutic privilege may apply; in these cases, a delay in diagnostic disclosure may be acceptable. Although the psychological risks tied to the disclosure of an AD diagnosis (Draper et al., 1998; Carpenter et al., 2008) will require further studies, it is wise to consider that symptoms of anxiety and depression may increase in people who receive a diagnosis of MCI due to AD. Diagnostic disclosure could cause a worsening in the patient’s quality of life that cannot be balanced by the respect for the patient’s autonomy. On the contrary, patients’ autonomy, i.e., the ability to manage their own life, could even be put at risk by the disclosure. Similarly, subjects without family support and those who lack close persons with whom to share information and disease experience are in a situation of frailty, and the risk that they will not to be able to cope with the disclosure is higher. To preserve their psychological and moral integrity, a delay in disclosure may be required. During this time, the patient is invited to involve a trusted person in the diagnostic disclosure, and additional psychological support is put in place.

Other patients who require a delay in diagnostic disclosure are those who clearly show that they do not wish to know the diagnosis. Their wish must always be respected, as it is the patient’s right – but not his/her duty – to be aware of information related to his/her health and life.

In all these cases, the physician, without deceiving the patient, will provide only a strict amount of information that the subject can cope with (Whitehouse et al., 2004). A delay in complete communication of the results is justified for several reasons.

First, the decision to not provide all the information to the patient at the time of the assessment does not preclude the possibility of further discussion between the physician and the patient, during which new elements can be added once the patient is ready to cope with the information. The nature of the diagnosis of MCI due to AD actually gives the physician the chance to review information over a period of time based on the patient’s condition and wishes. In the meantime, the subject’s features can also be better defined, still persisting a full patient’s competence. The physician will schedule frequent follow-up visits to be able to monitor the evolving situation. Moreover, it is important to stress that, due to the lack of effective treatments, the delay in delivering information does not delay the option of treatment for the subject.

### THE ROLE OF THE SUBJECT’S FAMILY

Within the context of diagnostic disclosure, the role of a patient’s family differs from country to country on the basis of local social structure and cultural heritage. In some countries, such as Italy (Pucci et al., 2003), family members usually play a very important role in the process of diagnostic disclosure to the point where they often ask to receive the diagnosis before their relative and to play an active role in the diagnostic disclosure to the patient. This clearly helps the physician in the difficult task of communicating bad news and, at the same time, provides the family members with the information they need to understand the disease, to plan for the patient’s care, and to cope with the psychological and physical burden caregivers must carry during the years of the patients’ cognitive and behavioral deterioration. Within the context of MCI due to AD, it is quite common for the patient to autonomously decide to attend the memory clinic alone. We suggest that physicians always explore the patient’s availability to involve a family member or a close friend in the diagnostic process whenever possible. This involvement can both provide the patient with the necessary support during and after the diagnostic disclosure and allow the family to understand the situation and assist in making lifestyle choices. For these reasons, an option for the patient to share the diagnostic disclosure with a family member should be included in the informed consent process.

A further option to delegate a family member to receive communication should be discussed, and the physician should explore the possibility of talking to a family member. There may be situations in which communicating the probable diagnosis to the subject at the time of assessment is not suggested, as in the case of patients who show symptoms of anxiety and depression or patients who do not wish to know, but knowledge of the examination results is important and useful for the care of the subject and for the family organization.

## CONCLUSION

We have discussed the ethical issues related to the diagnostic use of biomarkers during a time in which biomarkers are still being validated. After justifying the ethical legitimacy of implementing a research diagnostic protocol, we have suggested a guideline for informed consent and diagnostic disclosure, taking into consideration what is known and what is not known about the value of the biomarkers as well as the peculiar features of the disease and treatment options. The Alzheimer’s clinical community and scientific societies, as well as patients’ associations, may wish to heed these early suggestions and thoroughly discuss them with the aim of developing widely shared guidelines.

## AUTHOR CONTRIBUTIONS

Both the authors conceived the study and wrote the manuscript, and each author contributed to the paper utilizing their bioethical (Corinna Porteri) and scientific-clinical (Giovanni B. Frisoni) expertise.

## Conflict of Interest Statement

The authors declare that the research was conducted in the absence of any commercial or financial relationships that could be construed as a potential conflict of interest.
